# Dual targeting of BCL2 and MCL1 rescues myeloma cells resistant to BCL2 and MCL1 inhibitors associated with the formation of BAX/BAK hetero-complexes

**DOI:** 10.1038/s41419-020-2505-1

**Published:** 2020-05-05

**Authors:** Carolane Seiller, Sophie Maiga, Cyrille Touzeau, Céline Bellanger, Charlotte Kervoëlen, Géraldine Descamps, Laurent Maillet, Philippe Moreau, Catherine Pellat-Deceunynck, Patricia Gomez-Bougie, Martine Amiot

**Affiliations:** 1 CRCINA, INSERM, CNRS, Université d’Angers, Université de Nantes, Nantes, France; 2 SIRIC ILIAD, Angers, Nantes, France; 3 Service d’Hématologie Clinique, Unité d’Investigation Clinique, CHU, Nantes, France; 4 Plate-forme Therassay Onco-Hématologie, Capacités, Université de Nantes, Nantes, France

**Keywords:** Myeloma, Apoptosis

## Abstract

Multiple myeloma is a plasma cell malignancy that escapes from apoptosis by heterogeneously over-expressing anti-apoptotic BCL2 proteins. Myeloma cells with a t(11;14) translocation present a particular vulnerability to BCL2 inhibition while a majority of myeloma cells relies on MCL1 for survival. The present study aimed to determine whether the combination of BCL2 and MCL1 inhibitors at low doses could be of benefit for myeloma cells beyond the single selective inhibition of BCL2 or MCL1. We identified that half of patients were not efficiently targeted neither by BCL2 inhibitor nor MCL1 inhibitor. Seventy percent of these myeloma samples, either from patients at diagnosis or relapse, presented a marked increase of apoptosis upon low dose combination of both inhibitors. Interestingly, primary cells from a patient in progression under venetoclax treatment were not sensitive ex vivo to neither venetoclax nor to MCL1 inhibitor, whereas the combination of both efficiently induced cell death. This finding suggests that the combination could overcome venetoclax resistance. The efficacy of the combination was also confirmed in U266 xenograft model resistant to BCL2 and MCL1 inhibitors. Mechanistically, we demonstrated that the combination of both inhibitors favors apoptosis in a BAX/BAK dependent manner. We showed that activated BAX was readily increased upon the inhibitor combination leading to the formation of BAK/BAX hetero-complexes. We found that BCLXL remains a major resistant factor of cell death induced by this combination. The present study supports a rational for the clinical use of venetoclax/S63845 combination in myeloma patients with the potential to elicit significant clinical activity when both single inhibitors would not be effective but also to overcome developed in vivo venetoclax resistance.

## Introduction

The evasion of apoptosis is one of hallmarks of cancer, highlighting the important role of this pathway in survival of tumor cells. Apoptosis is under the control of BCL2 family proteins that behave as arbiters of cell fate and thus representing attractive therapeutic targets. Anti-apoptotic proteins prevent apoptosis by sequestering both BH3-only and effector pro-apoptotic proteins^1^. To overcome this resistance mechanism, significant advances have been made over the past few years towards the discovery of BH3-mimetics that inhibit these protein–protein interactions. BH3-mimetics targeting BCL2 (venetoclax)^2^ or MCL1(S64315, AMG176, AZD5991)^3–5^ are now clinically available.

Multiple myeloma is a bone marrow plasma cell malignancy that remains incurable. Myeloma cells escape to apoptosis by over-expressing anti-apoptotic BCL2 proteins allowing the sequestration of high levels of pro-apoptotic proteins. Consequently, myeloma cells are primed for death and present a particular vulnerability to BH3-mimetics. On one hand, venetoclax exhibits objective response in t(11;14) myeloma patients expressing low levels of BCLXL and MCL1 resistance factors^6,7^. On the other hand, MCL1 BH3-mimetics have demonstrated that a large proportion of myeloma cell lines and patient samples rely on MCL1 for survival^3,8,9^. Of note, MCL1 dependency was shown to increase at relapse^9^. Although a specific signature of MCL1 dependency has not been yet defined, different studies identified BCXL as the major resistance factor^3,4^. Interestingly, we recently identified a group of myeloma samples not sensitive to neither BCL2 nor MCL1 BH3-mimetics^9^.

The present study aimed to determine whether the combination of BCL2 inhibitor venetoclax and MCL1 inhibitor S63845 at low doses could be of benefit for myeloma cells beyond the single selective inhibition of BCL2 or MCL1, especially for myeloma cells not dependent on BCL2 or MCL1. In addition, the mechanism of action of the drug combination was studied with a particular focus on the implication of BH3-only proteins and activation of BAX and BAK effectors.

## Results

### Primary MM samples are mostly MCL1 dependent without influence of 1q amplification

**Fig. 1:**
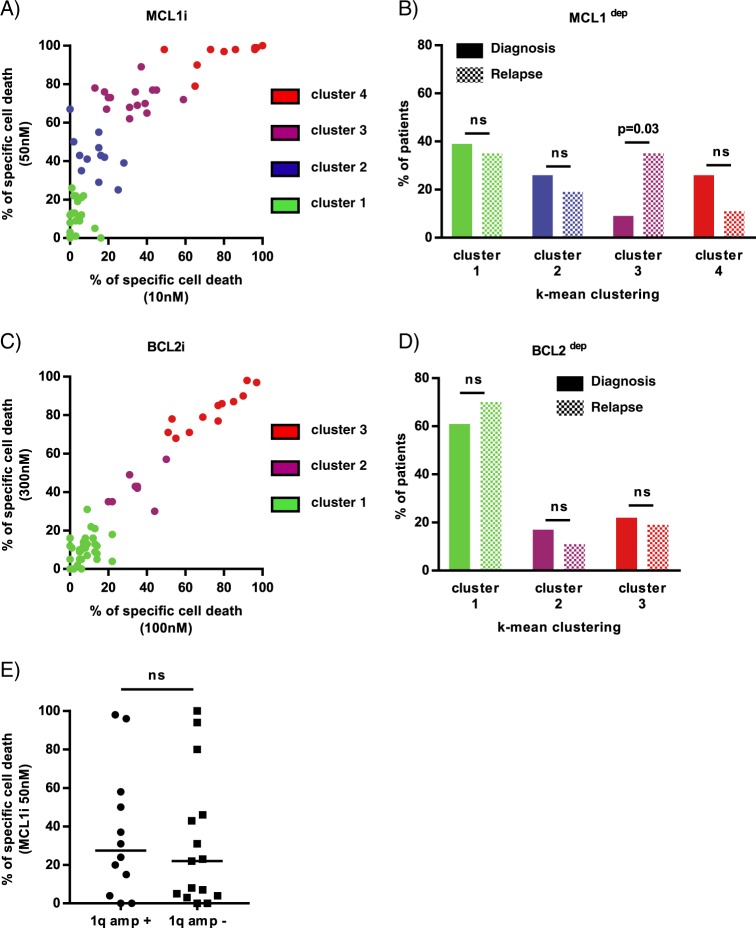
Primary MM cells are mainly MCL1 dependent without influence of 1q gain. Data clustering as assessed by k-means is shown for MCL1 and BCL2 inhibitors induced cell death in 60 patients (*n* = 1000 initiations of algorithm). **a** Clusters for MCL1 inhibitor response (S63845 12.5, 25 and 50 nM) with increasing sensitivity from 1 to 4. **b** Patient distribution according to disease status for MCL1 sensitivity clustering. Statistical analysis was done using Fisher’s exact test. **c** Clusters for BCL2 inhibitor response (venetoclax 100, 300, and 1000 nM) with increasing sensitivity from 1 to 3. **d** Patient distribution according to disease status for BCL2 sensitivity clustering. **e** Primary MM cells sensitivity to MCL1 inhibitor S63845 (50 nM) according to 1q gain (*n* = 27). 1q amplification was detected by FISH analysis. Statistical analysis was done using Mann–Whitney test, *p* = 0.64.

We determined the sensitivity to BCL2 and MCL1 inhibitors of 60 consecutive myeloma samples (23 at diagnosis and 37 at relapse) with a percentage of plasma cells of at least 3%. The definition of MCL1 and BCL2 dependency groups was achieved using an unbiased cell death clustering by k-means, as previously reported^9^. Thus, the optimal number of clusters was 4 for the MCL1 inhibitor and 3 for the BCL2 inhibitor with cluster 1 being the less sensitive and cluster 3 or 4 the more sensitive. The analysis of this cohort demonstrated that 63% of samples responded to MCL1 inhibition (cluster 2, 3, and 4) (Fig. 1a) while only 33% were sensitive to BCL2 inhibition (cluster 2 and 3) (Fig. 1c). The repartition of MCL1 and BCL2 dependent samples between diagnosis and relapse showed that the dependence on MCL1 significantly increased at relapse in the intermediate responder cluster 3 (from 9 to 35%, *p* = 0.03) (Fig. 1b), while the sensitivity to BCL2 inhibitor was independent of disease status (Fig. 1d), as previously shown^9^. Of interest, 1q amplification did not impact MCL1 dependency, as demonstrated in 27 patient samples (Fig. 1e).

### MCL1 and BCL2 inhibitor combination efficiently targets most primary cells resistant to single inhibitor

**Fig. 2:**
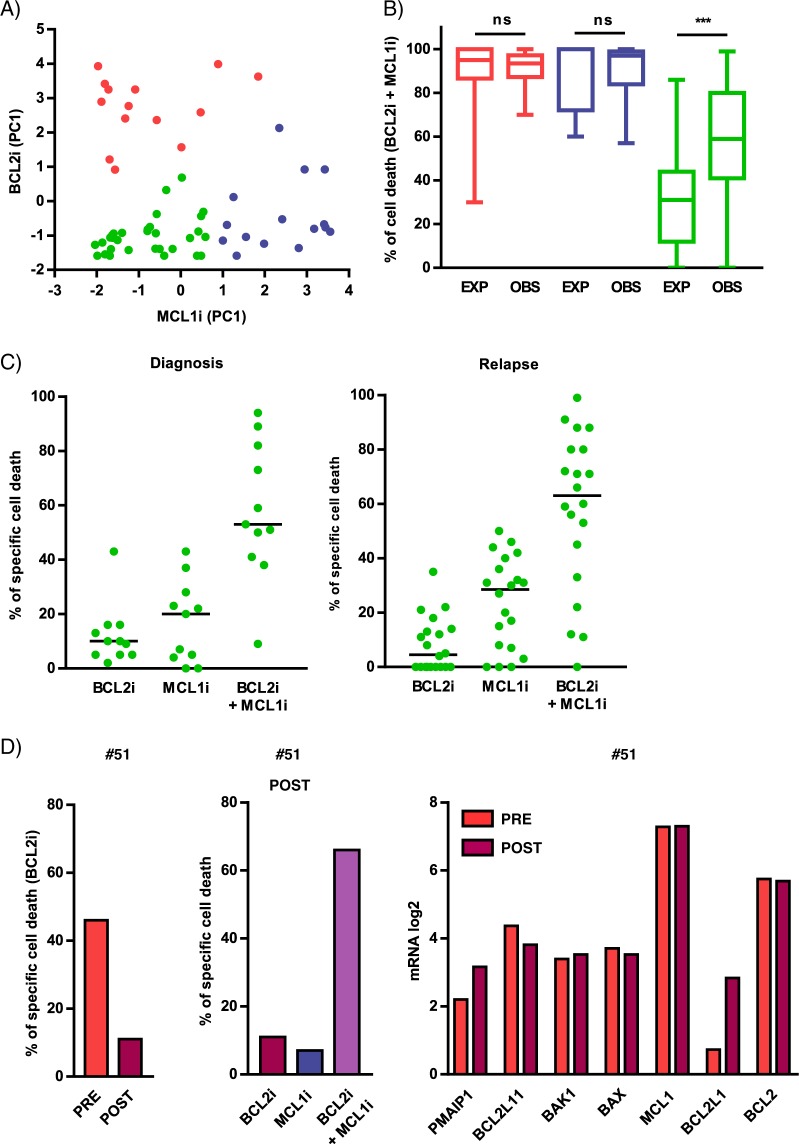
The combination of BCL2 and MCL1 inhibitors is efficient in a majority of primary cells resistant/poorly sensitive to each single inhibitor. **a** Novel unbiased cell death clustering by k-means in 60 patient’s samples combining cell death induced by S63845 (12.5, 25, and 50 nM) and venetoclax (100, 300, and 1000 nM) as single agents (*n* = 1000 initiations of algorithm). Three clusters of patient’s samples have been defined and corresponded to poorly sensitive/resistant to both venetoclax and S63845 (green, *n* = 31), venetoclax sensitive (red, *n* = 14) and S63845 sensitive (blue, *n* = 15). Data are represented according to each sample principal component (PC1) values for venetoclax and S63845 sensitivity. **b** Analysis of the sum of cell death induced by MCL1 + BCL2 inhibitors (expected) compared to cell death observed with the MCL1 and BCL2 inhibitors combination in the three clusters of patients. Values of MCL1 and BCL2 inhibitor combination are indicated Table 1. Statistical analysis was done using Wilcoxon test. **c** Cell death induced by MCL1 and BCL2 inhibitors combination in poorly sensitive/resistant MM patient samples (green cluster). Cell death induced by MCL1 inhibitor, BCL2 inhibitor and their combination in patient’s samples at diagnosis (*n* = 11) and relapse (*n* = 20). Optimal cell death threshold was set at 50%. **d** Analysis of primary MM cells from patient #51 before (PRE) and after (POST) venetoclax treatment. Left panel: comparison of ex vivo venetoclax (300 nM) sensitivity before (PRE) and after (POST) venetoclax treatment. Middle panel: Analysis of ex vivo venetoclax (300 nM), S63845 (25 nM) and venetoclax/S63845 combination sensitivity after venetoclax treatment. Right panel: comparison of BCL2 family expression analyzed by DGE-RNA seq sensitivity before (PRE) and after (POST) venetoclax treatment.

We studied whether low doses of BCL2 and MCL1 inhibitor combination could provide benefit beyond the effect of each single inhibitor. We performed a novel unbiased cell death clustering by k-means based on the response to MCL1 inhibitor in relation to the response to BCL2 inhibitor. As shown in Fig. 2a, three clusters were identified; namely, resistant/poorly responders to both inhibitors (green, *n* = 31), highly MCL1 dependent (blue, *n* = 15) and highly BCL2 dependent samples (red, *n* = 14) (Table 1). We next analyzed the effect of the MCL1 and BCL2 inhibitor combination in these three clusters. Figure 2b showed that resistant/poorly sensitive samples benefited the most from the combination with a synergistic effect of at least 2-fold increase of median cell death, (observed versus expected *p* < 0.0001). Indeed, in this group, a marked induction of apoptosis by the combination (>50%) was observed in 70% of samples either at diagnosis (8 out of 11) or at relapse (14 out of 20), confirming the interest of this combination for resistant/poorly responder patients (Fig. 2c). According to these results, we report the case of a 72 year-old man patient (#51) diagnosed with a symptomatic kappa light chain MM in 2015. Cytogenetic analysis revealed the presence of a t(11;14). He was treated with lenalidomide/dexamethasone regimen until disease progression in December 2016. At this time, he received venetoclax/dexamethasone in the context of a phase 2 trial. He achieved partial response and experienced disease progression on therapy after 6 months. The ex vivo testing of primary cells from this patient before venetoclax treatment reported 46% cell death induced by venetoclax (300 nM). Six months later, cell death induced by BCL2 inhibitor dropped to 11% while it was 7% upon MCL1 inhibitor S63845 (25 nM). Of interest, the combination of both inhibitors induced 66% cell death, indicating that the combination could overcome venetoclax resistance (Fig. 2d). 3′Digital Gene Expression (DGE) RNA-sequencing comparison analysis of samples from patient #51 at both time points demonstrated a readily increase in *BCL2L1* expression (3.9-fold increase) at the time of disease progression and ex vivo BCL2 resistance, while similar mRNA levels were observed for the other BCL2 members, either anti-apoptotics, effectors or BH3-only molecules.

**Table 1:** Sensitivity of 60 myeloma primary samples to BCL2 and MCL1 inhibitor combination.

#	Characteristics of patient				% of cell death (MCL1i + BCL2i)
	Age/sex	Disease	Status	% of PC	
1	58/F	MM	Diag	3%	78
2	70/F	MM	Diag	19%	85
3	73/M	MM	Diag	9%	88
4	59/M	MM	Diag	7%	93
5	70/M	MM	Diag	12%	97
6	65/M	MM	Diag	4%	99
7	72/M	MM	Rel	6%	70
8	62/M	MM	Rel	13%	89
9	71/M	MM	Rel	5%	92
10	61/F	MM	Rel	13%	94
11	45/F	MM	Rel	4%	96
12	53/M	MM	Rel	22%	97
13	69/M	MM	Rel	37%	98
14	69/F	MM	Rel	4%	100
15	61/M	MM	Diag	9%	84
16	59/M	MM	Diag	6%	95
17	74/M	MM	Diag	20%	98
18	55/M	MM	Diag	21%	99
19	56/M	MM	Diag	39%	99
20	76/M	MM	Diag	18%	99
21	65/M	MM	Rel	18%	57
22	65/F	MM	Rel	5%	63
23	58/M	MM	Rel	4%	84
24	61/F	MM	Rel	4%	89
25	66/F	sPCL	Rel	12%	97
26	83/F	MM	Rel	9%	97
27	62/F	MM	Rel	19%	98
28	60/M	MM	Rel	8%	100
29	70/F	MM	Rel	18%	100
30	76/M	MM	Diag	3%	9
31	88/F	MM	Diag	4%	38
32	82/F	MM	Diag	3%	41
33	73/F	MM	Diag	17%	50
34	55/F	MM	Diag	8%	51
35	66/F	MM	Diag	21%	53
36	71/F	MM	Diag	23%	59
37	56/F	MM	Diag	22%	73
38	67/F	MM	Diag	6%	82
39	66/M	MM	Diag	15%	89
40	54/F	MM	Diag	43%	94
41	58/F	sPCL	Rel	80%	0
42	60/F	MM	Rel	10%	11
43	83/M	MM	Rel	14%	12
44	81/F	sPCL	Rel	22%	22
45	63/F	MM	Rel	20%	33
46	81/M	MM	Rel	9%	45
47	72/M	MM	Rel	26%	53
48	70/M	MM	Rel	4%	56
49	76/M	MM	Rel	8%	59
50	78/M	MM	Rel	11%	60
51	72/M	MM	Rel	59%	66
52	63/F	MM	Rel	21%	71
53	70/M	sPCL	Rel	8%	71
54	65/M	MM	Rel	3%	72
55	78/M	MM	Rel	4%	80
56	74/F	MM	Rel	7%	80
57	64/M	sPCL	Rel	31%	88
58	86/F	MM	Rel	76%	88
59	82/M	sPCL	Rel	23%	91
60	76/M	MM	Rel	7%	99

The percentage of cell death induced by the BCL2 and MCL1 inhibitor combination (300 nM of venetoclax with 25 nM of S63845 or 2.5 μM of A1210477) was indicated. Patients have been ordered according to clustering defined in Fig. 2a: patients #1 to #14 were highly BCL2 dependent (red cluster); patients #15 to # 29 were highly MCL1 dependent (blue cluster) and patients #30 to # 60 were resistant or poorly sensitive to MCL1, BCL2, or both inhibitors (green cluster).

*F* female, *M* male, *MM* multiple myeloma, *sPCL* secondary plasma cell leukemia, *Diag* diagnosis, *Rel* relapse, *PC* plasma cells.

### Combined targeting of BCL2 and MCL1 induced apoptosis in a synergistic manner in myeloma cell lines resistant to BCL2 and MCL1 inhibitors

**Fig. 3:**
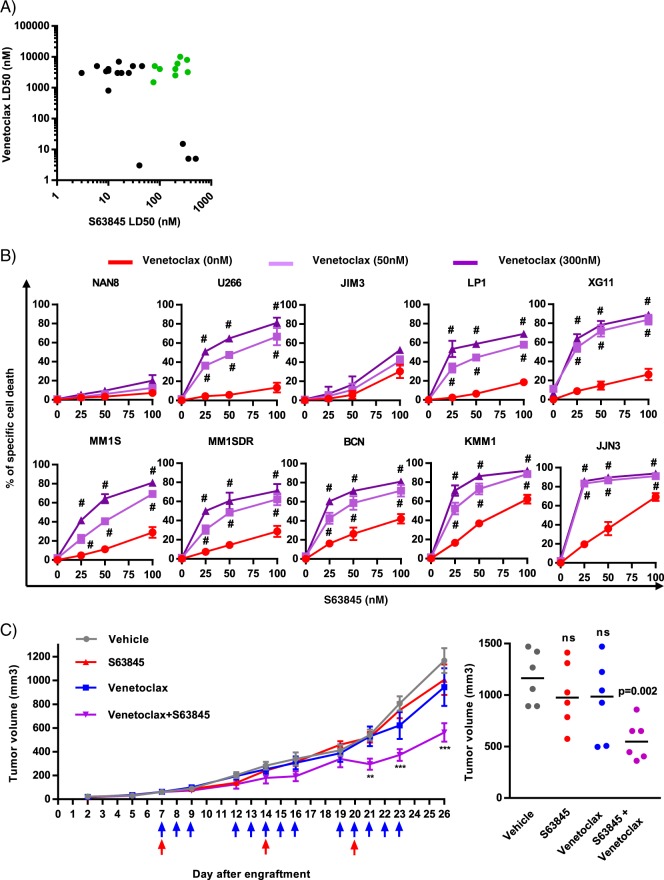
The combination of BCL2 and MCL1 inhibitors is effective and synergic in HMCLs resistant to each inhibitor alone. **a** Sensitivity of 26 HMCLs to S63845 versus venetoclax. After 24 h of treatment with increasing concentrations of S63845, cell death was assessed by Annexin V staining and LD50s were calculated from at least three independent experiments. Venetoclax LD50s were previously established^9^. HMCLs resistant to both BH3-mimetic are indicated in green. **b** JJN3, KMM1, BCN, MM1S, MM1SDR, XG11, LP1, JIM3, U266, and NAN8 were treated with increasing doses of the combination S63845/venetoclax for 24 h. Cell death was assessed by Annexin V staining. Data represent the mean of three independent experiments ± SD. Combination Index (CI) was calculated with Compusyn software, Hash represents CI < 0.4. **c** In vivo effect of S63845/venetoclax on tumor growth in U266 xenograft model. U266 xenografts were treated with vehicle (p.o. and i.v.), venetoclax (p.o.) (blue arrows), S63845 (i.v.) (red arrows) or venetoclax (p.o.) + S63845 (i.v.) (violet arrows) as indicated. Left panel: tumor growth was monitored by measurement of tumor volumes. Mean tumor volume ± SEM of each treatment group (six mice per group) is depicted. Statistical analysis was performed using a two-way ANOVA test, followed by a Tukey’s post-test (*p < 0.05, ***p < 0.001). Right panel: analysis of tumor volumes at day 26. Mann–Whitney test was used for statistical analysis.

The sensitivity to S63845 and venetoclax was also analyzed in a panel of 26 HMCLs. In agreement with previous studies^3,8^, we found that a large proportion of myeloma cell lines (62%) exhibited high (LD50 < 50 nM) or intermediate (LD50 < 120 nM) sensitivity to S63845 (Fig. 3a, Supplementary Table S1). According to our previous study^6^, only a restricted subgroup of HMCLs harboring the t(11;14) translocation was efficiently killed by venetoclax (Fig. 3a). In agreement with primary sample findings, we identified a sub-group of HMCLs (green cluster *n* = 10) strongly resistant to venetoclax and either poorly sensitive to S63845 (KMM1, JJN3) or totally resistant to S63845 (U266, LP1, MM1S, MM1SDR, NAN8, BCN, JIM3, XG11) (Fig. 3a). Venetoclax/S63845 combination assessed at low doses was effective and synergistic in most cell lines (combination index < 0.4) including the MM1SDR cell line with the exception of NAN8 and JIM3 HMCL (Fig. 3b). Resistance of these latter cell lines was not related to mutations in neither *BCL2* nor *MCL1* genes, as demonstrated in our previous work^10^.

### BCL2 and MCL1 inhibitor combination is efficient in U266 xenograft model

To address the efficacy of inhibitor combination in vivo, we injected U266 HMCL resistant to both inhibitors in NSG mice. Mice harboring subcutaneous U266 xenografts were treated with S63845, venetoclax or the combination of S63845/venetoclax as indicated in Fig. 3c. While venetoclax or S63845 treatment had no effect on tumor growth, the combination of both BCL2 and MCL1 inhibitors significantly delayed tumor growth (Fig. 3c). Indeed, at 26 days a significant retard of tumor growth was observed in five out of six mice treated by the combination (Fig. 3 right panel). No significant body weight loss was observed under the combination treatment (Supplementary Fig. S1).

### The BCL2 and MCL1 inhibitor combination induced apoptosis in myeloma cells resistant to each inhibitor through the formation of the BAK/BAX hetero-complexes

**Fig. 4:**
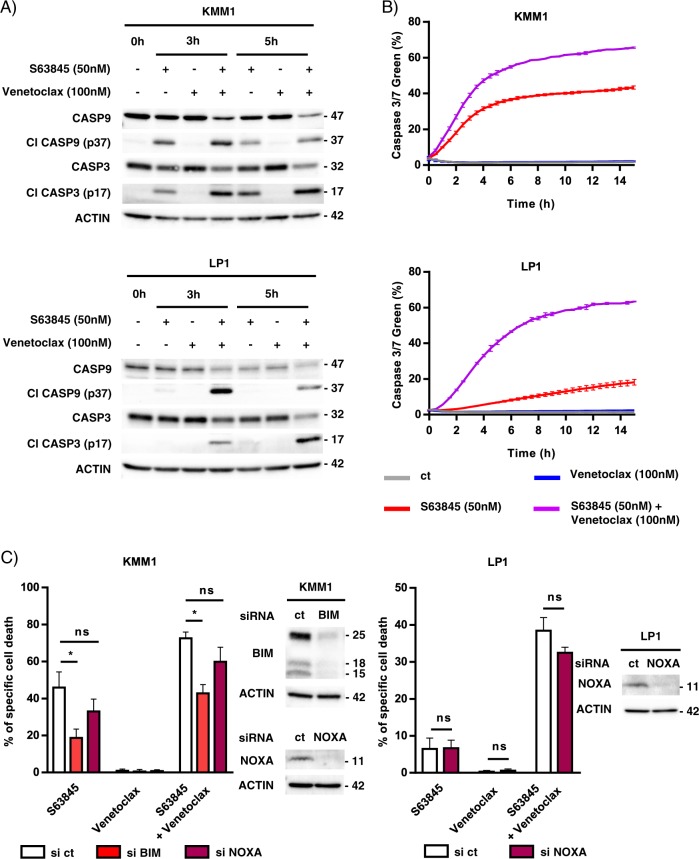
The combination of BCL2 and MCL1 inhibitors induced early hallmarks of apoptosis in HMCLs. **a** Immunoblot of caspase 9 and 3 activation in KMM1 and LP1 HMCLs after 3 and 5 h of S63845 and/or venetoclax treatment. **b** Caspase activation was monitored by live-cell imaging using IncuCyte S3 during 15 h exposure to S63845 (50 nM) and/or venetoclax (100 nM) and in the presence of a Caspase 3–7 dye. Results represent the mean ± SD of two independent experiments. **c** Transfection of KMM1 and LP1 HMCLs with scramble, BIM or NOXA siRNA for 48 h followed by 24 h of S63845 (50 nM) and/or venetoclax (100 nM). Cell death was assessed by Annexin V staining. Silencing efficacy was validated by immunoblotting. Results represent the mean ± SD of five independent experiments. Mann–Whitney test was used for statistical analysis (*p < 0.05).

**Fig. 5:**
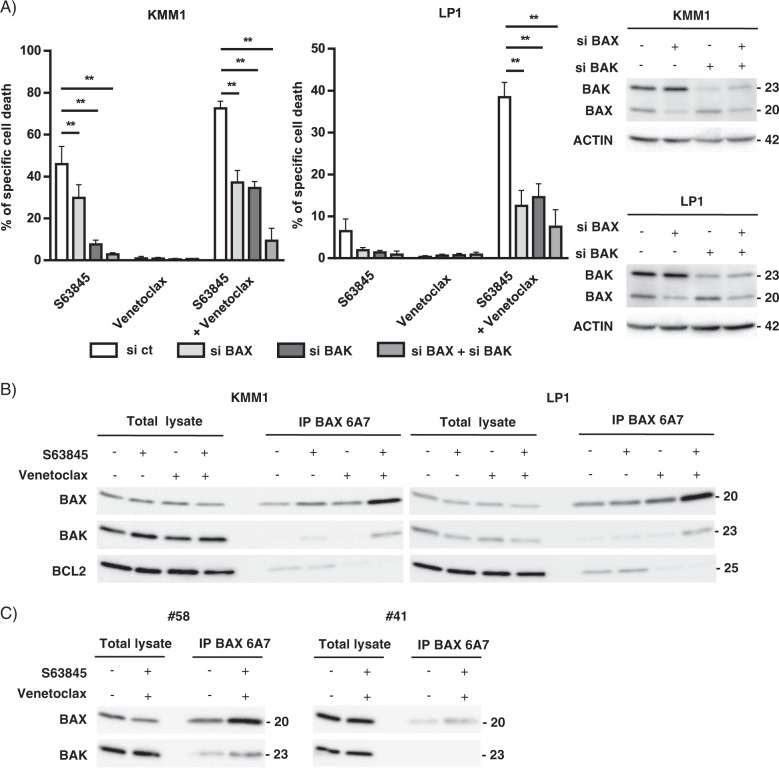
BAX and BAK are essential for S63845/venetoclax combination response through the BAX/BAK heterocomplex formation. **a** KMM1 and LP1 HMCLs were transfected with scramble, BAX and/or BAK siRNA for 48 h followed by 24 hours of S63845 and/or venetoclax. Results represent the mean ± SD of five independent experiments. Mann–Whitney test was used for statistical analysis (*p < 0.05, **p < 0.01). **b** HMCLs were treated with S63845 (25 nM for KMM1, 100 nM for LP1), venetoclax (100 nM) or a combination of both during 3 h. Immunoprecipitation reactions were performed overnight using the 6A7 BAX-Agarose coupled mAb. Immunoprecipitation eluates were analyzed by western blot. **c** CD138 purified primary cells were treated or not with the S63845 (25 nM)/venetoclax (100 nM) combination during 3 h and processed as in **b**.

Caspase activation was investigated in KMM1 and LP1 HMCLs both by western blotting analysis and live-cell imaging. The cleavage of caspase 9 and 3 was observed as soon as 3 h of treatment, the activated form of caspase 3 progressed at 5 h in both cell lines (Fig. 4a). The activation of caspases 9 and 3 was also found upon S63845 alone in KMM1 cells, according to its intermediate sensitivity to the MCL1 inhibitor. In addition, a kinetic study of caspase 3/7 activation was performed by live-cell imaging during 15 h of S63845 and/or venetoclax treatment (Fig. 4b). Caspase 3/7 activation induced by each BH3-mimetic alone agreed with the respective sensitivity of KMM1 and LP1. Of note, the kinetic of caspase activation upon the S63845/venetoclax combination was faster in KMM1 than LP1 yet both reached a plateau characterized by 66% of KMM1 and 63% of LP1 caspase 3/7 positive cells. These results suggest that the apoptotic machinery may not be uniform in a cell population as previously reported^11^. Others and us have demonstrated that BH3-mimetics induce the release of pro-apoptotic proteins, BH3-only and multi-domain effectors, from their anti-apoptotic targets^4,12,13^. Therefore, we first studied the contribution of BH3-only proteins to the apoptotic response induced by the S63845/venetoclax combination. As shown in Fig. 4c, BIM silencing in KMM1 cells significantly decreased the cell death response induced by both S63845 alone (57% decrease, *p* = 0.0159) and also by the S63845/venetoclax combination (42% decrease, *p* = 0.0159). However, this protection was not complete. The knock-down of NOXA, which interacts exclusively to MCL1, did not impact this cell death response neither in KMM1 nor in the BIM-deleted LP1 cells. We further studied the contribution of BAX and BAK in cell death induced by the S63845/venetoclax combination. While the knock-down of each effector partially suppressed the cell death induced by the BH3-mimetic combination in both KMM1 (40% and 55% inhibition for BAX and BAK silencing, respectively) and LP1 cells (68% and 56% inhibition for BAX and BAK, respectively), the simultaneous BAX/BAK silencing almost completely suppressed apoptosis in both cell lines (88% inhibition for KMM1 and 76% inhibition for LP1) (Fig. 5a). In agreement with our previous studies, BAK silencing was more protective (87% of cell death inhibition) than BAX (22% of cell death inhibition) against S63845 in KMM1. We next analyzed the activation status of BAX using the conformational active antibody anti-BAX 6A7. To this aim, KMM1 and LP1 cells were treated with the respective BH3-mimetic for 3 h and immunoprecipitations were performed using the 6A7 BAX antibody. As shown in Fig. 5b, we found a small pool of activated BAX in MM untreated viable cells, which slightly increased under each individual BH3-mimetic. Interestingly, activated BAX was readily increased upon the inhibitor combination and formed heterocomplexes with BAK, as shown by co-immunoprecipitation in both cell lines (Fig. 5b). Additionally, activated BAX formed complexes with BCL2 in untreated viable cells, which were disrupted under venetoclax treatment. No interaction between activated BAX and MCL1 was detected (data not shown). Interestingly, the presence of activated BAX was readily detected in primary cells from a sample that responded to the BH3 combination (#58, 88% of cell death) (Fig. 5c, left panel) and also present, in a lesser extent, in primary cells from non-responder sample (#41, 0% of cell death) (Fig. 5c, right panel). However, the presence of heterocomplexes of activated BAX with BAK was only observed upon the BH3-mimetic combination in primary cells from the responder patient. These results suggested that apoptosis commitment upon the S63485/venetoclax combination was associated with the early formation of BAX/BAK heterocomplexes, which seemed to be a common feature of this cell death response observed not only in two HMCLs but also in primary cells from a MM patient.

### BCLXL remains the major resistant factor of BCL2 and MCL1 inhibitor combination induced apoptosis

**Fig. 6:**
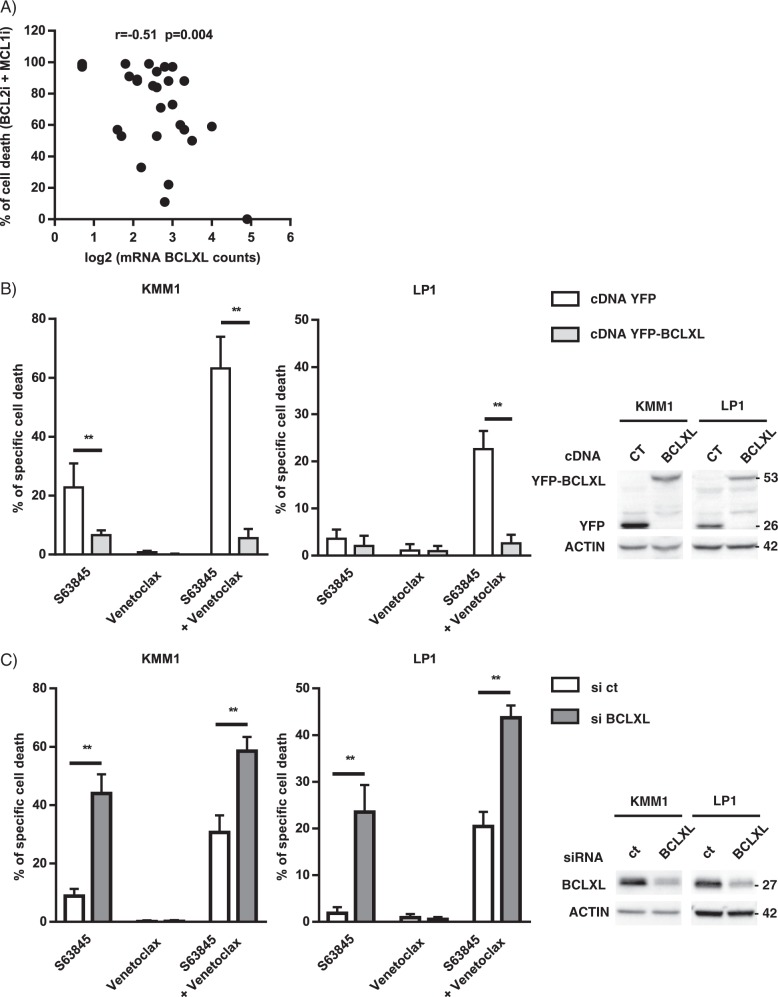
BCLXL contributes to the resistance to the S63845/venetoclax combination. **a** Correlation of *BCLXL* expression and the response to the combination of BCL2 and MCL1 inhibitors. Total RNA was obtained from purified CD138^+^ myeloma cells from 29 samples and the expression of BCLXL was analyzed by DGE seq. Log2 normalized BCLXL mRNA counts were plotted against the percentage of cell death induced by the BCL2 and MCL1 inhibitor combination. Spearman correlation is indicated. **b** KMM1 and LP1cells were transfected during 24 h with control cDNA (YFP) or BCLXL cDNA (YFP-BCLXL) followed by 24 h of S63845 (50 nM) and/or venetoclax (100 nM) treatment. Cell death was assessed in YFP positive cells by Annexin V staining. Results represent the mean ± SD of five independent experiments, (*p < 0.05). Mann–Whitney test was used for statistical analysis. Overexpression of YFP and YFP-BCLXL was validated by immunoblotting. **c** Transfection of KMM1 and LP1 cells with scramble or BCLXL siRNA for 48 h followed by 24 h of S63845 (12.5 nM) and/or venetoclax (100 nM) treatment. Cell death was assessed by Annexin V staining. Results represent the mean ± SD of five independent experiments. Mann–Whitney test was used for statistical analysis (*p < 0.05). Silencing efficacy was validated by immunoblotting.

Because we have previously shown that *BCL2L1 (BCLXL)* is involved in the resistance to both BCL2 and MCL1 inhibitors, we analyzed the expression of *BCL2L1* by DGE RNA-sequencing (Supplementary Table S2). Among the 60 MM samples analyzed for the response to BCL2 and MCL1 inhibitor combination, 29 samples were purified using CD138 mAb and processed for digital gene expression profiles. We found that *BCL2L1* expression inversely correlated with the response to the BH3-mimetic combination (*r* = 0.51 *p* = 0.004) (Fig. 6a). These results strongly suggest that a high level of BCLXL remains a resistance factor for the BH3-mimetic combination. To further examine the role of BCLXL in the induction of cell death by the S63845/venetoclax combination, BCLXL was either over-expressed or silenced in HMCLs. To over-express BCLXL, YFP-fused *BCLXL* was transiently transfected in both KMM1 and LP1 cells and BCXL over-expression was followed by the analysis of YFP‐positive cells (Supplementary Fig. S2). As shown in Fig. 6b, BCLXL over-expression induced a 90% and 87% decrease of apoptosis induced upon S63845/venetoclax combination in KMM1 and LP1, respectively. As expected, BCLXL over-expression was also protective against S63845 alone in KMM1 cells. To further substantiate the implication of BCLXL as a major resistance factor, we demonstrated that the efficient silencing of BCLXL resulted in a 2-fold increase of cell death induced upon S63845/venetoclax combination, in both KMM1 and LP1 cells (Fig. 6c). A similar sensitization was observed in cell death response induced by S63845 but not by venetoclax alone (Fig. 6c).

## Discussion

The particular efficacy of venetoclax in the t(11;14) myeloma samples was first demonstrated by ex vivo testing of myeloma cell sensitivity and then confirmed by the clinical trial evaluating venetoclax monotherapy in refractory/relapsed myeloma^6,7,14^ and compassionate assays^15,16^. Of interest, a recent study extended these initial findings showing that functional testing of venetoclax sensitivity predicts clinical response in myeloma^17^. Altogether, these results provide the proof of concept that ex vivo testing currently represents the best way to guide the use of BH3-mimetics for myeloma treatment in the absence of appropriate molecular biomarkers. As MM is particularly heterogeneous with respect to anti-apoptotic protein dependencies, the identification of individual dependence remains the prerequisite for all studies including BCL2 family inhibitors. The cohort of patients described in this study and the HMCL collection confirmed previous findings^18^, indicating that a large proportion of myeloma cells are MCL1 dependent, particularly at relapse, while BCL2 dependence is more restricted. Our analysis on 27 patient samples demonstrated that 1q amplification had no impact on the sensitivity to MCL1 inhibitor. This result is in line with the absence of correlation between the level of expression of *MCL1* and the sensitivity to MCL1 inhibitor S63845 reported in different hematological malignancies^3,4,9^. Altogether these findings contradict a recent study reporting that patients with 1q amplification expressed higher level of MCL1 and were more sensitive to MCL1 inhibitor^18^. In our study, myeloma cells poorly responding to both BCL2 and MCL1 inhibitors represented around 50% of patient samples, which could be found either at diagnosis or relapse. As BH3-mimetic combination therapies could help to overcome resistance to single agent treatment, we first demonstrated that the group of resistant/poorly responder samples to BCL2 and MCL1 inhibitors was the group of patients taking the strongest advantage of the combination with a synergistic ex vivo effect. Thus, we focused on this group of patients. Because the use of BCL2 and MCL1 inhibitor combination could also increase toxicities, we paid attention to evaluate suboptimal doses of each inhibitor in myeloma cells. Indeed, combination of low nanomolar doses of both inhibitors, ineffective as single agents, had strong efficacy (50% cell death or more) in 70% of samples independently of disease status. In addition, we found that S63845/venetoclax combination exhibited marked anti-tumor activity in the U266 xenograft model resistant to both inhibitors, in agreement with studies that report efficacy of venetoclax with MCL1 inhibitors in myeloma models^19,20^. However, these previous studies mostly evaluate the effect of the BH3-mimetic combination on myeloma cells either sensitive to venetoclax or MCL1 inhibitors and consequently used different concentration of inhibitors according to the initial sensitivity of myeloma cells to each inhibitor. In particular, Siu et al. lately demonstrated the efficacy of combining AZD5991 with venetoclax in MM cells, yet authors stated that concentrations of venetoclax used in this study were relatively high^20^. Of note, the different xenograft models are not pertinent to address the safety of the combination since S63845 has a weak affinity for murine MCL1. We also reported a transient efficacy of the venetoclax/dexamethasone treatment in a t(11;14) MM patient with a subsequent emergence of resistance after 6 month treatment. For the first time we suggested that developed resistance to venetoclax could be due to the observed overexpression of *BCLXL*. Accordingly, it was recently demonstrated that venetoclax resistance was associated with increased BCLXL level in a CLL patient^21^. We demonstrated that the ex vivo combination therapy could overcome developed resistance to venetoclax associated with BCLXL increase. One may hypothesize that the combination of inhibitors can overcome the resistance due to BCLXL overexpression by inducing a massive release of pro-apoptotic proteins from MCL1 and BCL2, which may overwhelm the recapture capacity of BCLXL. Furthermore, the involvement of BCLXL in the resistance to MCL1 and to BCL2 inhibitors was previously reported in MM^7,9^ and in other cancers^4,22^. Our results demonstrated that the modulation of BCLXL can significantly affect cell death induced by the BH3-mimetic combination, yet the sole BCLXL level of expression seems not enough to predict the response to this combination.

The apoptotic response of the combination was caspase and BAX/BAK dependent, as already reported for the dual targeting of BCL2/MCL1 in AML^23^. Interestingly, we demonstrated that activated BAX was able to form heterocomplexes with BAK upon venetoclax/S63845 combination; this was a feature of cell death induced under this condition. Interestingly, BAX/BAK heterocomplexes were also detected on primary cells from a sensitive sample, suggesting that the interaction between activated BAX and BAK is a common mechanism of apoptosis induced by the simultaneous inhibition of BCL2 and MCL1. The absence of this complex in primary resistant cells supported this notion. Our results support a cooperative binding between BAX and BAK for the formation of mitochondrial membrane pores under the BH3-mimetic combination. BAX/BAK complex was already reported in other apoptotic responses, as in HeLa cells treated with TNF-α and in Burkitt’s lymphoma cells upon verotoxin-1 treatment^24,25^. These findings suggest that the interaction between activated BAX and BAK is involved in apoptosis triggering not only under the simultaneous inhibition of BCL2 and MCL1 but also upon other apoptotic signals. Although BAX and BAK may be individually capable to form pores at the mitochondria, it was shown that BAX may require BAK for optimal pore formation^26^. Finally, the rational combination using MCL1 and BCL2 inhibitors may hold the key to maximize the impact of MCL1 and BCL2 inhibition guided by ex vivo testing in patients not responding to BH3-mimetic monotherapy.

## Materials and methods

### Human myeloma cell lines and primary myeloma cells

Human myeloma cell lines (HMCLs) (*n* = 26) and MM1S Dexamethasone resistant cell line (MM1SDR) were characterized as previously described^27,28^. The XG1, XG2, XG3, XG5, XG6, XG10, XG11, NAN1, NAN3, NAN8, NAN11, MDN, and BCN HMCLs were derived in our laboratory. The KMS12PE and KMM1 HMCLs were kindly provided by Dr. Otsuki (Kawasaki Medical School, Kurashiki, Japan); JJN3, by Dr. Van Riet (Vrije Universiteit Brussel, Brussels, Belgium); JIM3, by Dr. MacLennan (Birmingham Medical School, Birmingham, UK); KARPAS-620, by Dr. Karpas (Cambridge Clinical School, Cambridge UK); and MM1S, by Dr. S. Rosen (Northwestern University, Chicago, USA). The AMO1, LP1, L363, NCI-H929, U266, OPM2 and SKMM-2 HMCLs were from DSMZ (Braunsweig, Germany). After obtaining informed consent, bone marrow/blood samples from MM patients were collected at the Department of Hematology, University Hospital of Nantes (MYRACLE study; NCT03807128).

### Reagents and antibodies

Venetoclax (ABT-199) and A1210477 were from Selleck Chemicals (Houston, TX, USA); S63845 from Chemietek (Indianapolis, USA).

The following antibodies were used: BCL2 (Dako, Cat.No.: M0887), BCLXL (Cell signaling, Cat.No.: 2764S), MCL1 (Santa Cruz Biotechnology, Cat.No.: sc-958), BAX (Enzo Life Sciences, Cat.No.: ADI-AAM-140), BAK (BD Biosciences, Cat.No.: AMO3), BIM (Millipore, Cat.No.:AB17003), NOXA (Enzo Life Sciences, Cat.No.: ALX-804-408-C100), CASPASE3 (Santa Cruz Biotechnology, Cat.No.: sc-7272), CASPASE9 (Santa Cruz Biotechnology, Cat.No.: sc-17784), cleaved CASPASE9 (Cell signaling, Cat.No.: 95015); ACTIN (Millipore, Cat.No.: MAB1501), GFP pAb (Abcam, Cat.No.: ab290).

Scramble, *BCLXL, BAX, BAK* and *NOXA* siRNAs were from Dharmacon. BIM siRNA from Santa Cruz Biotechnology.

### Cell death assays in myeloma cell lines (HMCLs) and primary cells

Cell death in HMCLs and HMCLs transfected with *YFP* vectors was determined using AnnexinV-FITC or AnnexinV-APC (Beckman Coulter), respectively. When statistical analyses were performed, cell death assays in HMCLs were independently replicated five times, reported as mean ± SD and analyzed with Mann–Whitney tests. Myeloma cells were identified using CD138 staining, cell dependency to anti-apoptotic BCL2 proteins was measured by the loss of CD138 staining as previously described^9^. Cell death assay of primary myeloma cells was carried out using mononuclear cells cultured with or without BH3 inhibitors. Venetoclax was tested at 100, 300, and 1000 nM while S63845 was evaluated at 10, 25, and 50 nM. In some MM samples, A1210477 was also tested at 2.5 μM.

### Analyses of cell dependencies in primary cells

Analyses were conducted under R-3.5.0 environment. To establish groups of dependence in an unbiased way, clustering of patients’ sensitivity to venetoclax and S63845 were performed as follows. Firstly, the optimal number of clusters was determined by the elbow method of the within-cluster sum of square with *k*-means clustering (2 ≤ *k* ≤ 4) using the Nbclust package^29^. Secondly, an iterative *k*-means procedure was performed on dataset with 1000 initiations. Because no Lethal-Dose-50 value could be calculated for each patient sample, we constructed the Principal Component 1 (PC1) value for each drug i.e., the coordinate of the sample on the 1st axis of the considered drug.

### IncuCyte S3 live-cell analysis

KMM1 or LP1 cells were plated into 96-well plates and treated with S63845 (50 nM) and/or venetoclax (100 nM) in the presence of Caspase-3/7 Green Apoptosis Assay Reagent (Essen BioScience) according to the manufacturer’s instructions. Culture plates were placed into the IncuCyte S3 live-cell analysis system and three fields per well were scanned every 30 min during 15 h. Caspase -3/7 green reagent fluorescence was normalized to cell confluence.

### Immunoblotting and Immunoprecipitation

Western blotting and immunoprecipitation reactions were performed as previously described^30^. Immunoprecipitations were performed using the anti-BAX 6A7-agarose (Santa Cruz Biotechnology, Cat.No.: sc-23959AC). Immunoprecipitations using lysates from purified primary CD138 positive cells were processed similarly.

### Gene expression analysis

Total RNA was obtained from purified CD138 + plasma cells using RNeasy mini kit (Qiagen) according to the manufacturer’s instructions. The mRNA expression of BCL2 family members was performed by 3′digital gene expression (DGE) RNA-sequencing protocol according to Kilens et al.^31^. Briefly, the libraries were prepared from 10 ng of total RNA from purified plasma cells. The mRNA poly(A) tail was tagged with universal adapters, well-specific barcodes and unique molecular identifiers (UMIs) during template-switching reverse transcriptase. Barcoded cDNAs from multiple samples were then pooled, amplified and tagmented using a transposon-fragmentation approach, which enriches for 3’ends of cDNA. A library of 350–800 bp was run on an Illumina HiSeq 2500 using a HiSeq Rapid SBS Kit v2 (50 cycles; FC-402-4022) and a HiSeq Rapid PE Cluster Kit v2 (PE-402-4002). Read pairs used for analysis matched the following criteria: all sixteen bases of the first read had quality scores of at least 10 and the first six bases correspond exactly to a designed well-specific barcode. The second reads were aligned to RefSeq human mRNA sequences (hg19) using bwa version 0.7.17. Reads mapping to several transcripts of different genes or containing more than three mismatches with the reference sequences were filtered out from the analysis. Digital gene expression profiles were generated by counting the number of unique UMIs associated with each RefSeq genes, for each sample.

### Transient transfection

KMM1 and LP1 myeloma cells were transfected with 100 pmol siRNA using RNAimaxTM Reagent (Life Technologies) or 0.2 µg cDNA using Lipofectamine 2000 (Life Technologies) according to the manufacturer’s instructions; cells transfected with siRNA or cDNA were treated with BH3-mimetics after 48 h and 24 h, respectively. *YFP* cDNA (*pEYFP-C1*) from BD Biosciences was used to express YFP-BCLXL protein as previously described^32^.

### In vivo xenograft studies

Animal studies were approved by the Committee on the Ethics of Animal Experiment (CEEA-Pdl06). The minimal number of animals needed for carrying out a comparative study (six mice per group) was chosen to respect the 3Rs. Six-week-old female NSG mice purchased from Charles River Laboratory (Saint Germain Nuelles, France) were inoculated subcutaneously into the right flank with 10^7^ U266 cells in 100 μl of PBS. Tumor volume was measured using a caliper and calculated as the length × width × width/2 (mm3). When tumor volumes reached ~40–100 mm^3^, mice were randomized into four groups (six mice per group) so that all groups contained mice with comparable calculated tumor volumes and treatments were initiated. Drugs administration was performed by oral gavage for venetoclax (100 mg/kg, InvivoChem, Libertyville, USA) 5 days a week and i.v. for S63845 (25 mg/kg, InvivoChem) every 6 days. Due to the color of used drugs, a blinding was not possible but experiments were performed by a neutral investigator.

## Supplementary information

Supplemental figure legends

Supplementary Table 1

Supplementary Table 2

Supplementary Figure 1

Supplementary Figure 2
