# Supplementary material for: Dual targeting of BCL2 and MCL1 rescues myeloma cells resistant to BCL2 and MCL1 inhibitors associated with the formation of BAX/BAK hetero-complexes
Source: Cell Death Dis. 2020 May 5;11(5):316. doi: 10.1038/s41419-020-2505-1 (PMC7200824; doi:10.1038/s41419-020-2505-1)
Supplement: Supplementary file 1 — Supplemental figure legends [file 41419_2020_2505_MOESM1_ESM.docx]

**Supplementary Figure 1**. In vivo effect of S63845/Venetoclax on body weight of mice grafted with U266 cells. U266 xenograft were treated with vehicle (p.o. and i.v.), venetoclax (p.o.) (blue arrows), S63845 (i.v.) (red arrows) or Venetoclax (p.o.) + S63845 (i.v.). as indicated. Mean body weight ± SEM of each treatment group (6 mice per group) are depicted.

**Supplementary Figure 2.** Flow cytometry dot plot representing Annexin V staining and YFP fluorescence in KMM1 24h after transfection of indicated cDNA.
