# Supplementary material for: Dual targeting of BCL2 and MCL1 rescues myeloma cells resistant to BCL2 and MCL1 inhibitors associated with the formation of BAX/BAK hetero-complexes
Source: Cell Death Dis. 2020 May 5;11(5):316. doi: 10.1038/s41419-020-2505-1 (PMC7200824; doi:10.1038/s41419-020-2505-1)
Supplement: Supplementary file 2 — Supplementary Table 1 [file 41419_2020_2505_MOESM2_ESM.docx]

Supplementary Table 1

| Myeloma cell lines characteristics | | | Venetoclax  LD_50_ | S63845  LD_50_ |
| --- | --- | --- | --- | --- |
| MDN | t(11;14) | TP53^WT^ | 3 | 40 |
| KARPAS620 | t(11;14) | TP53^mut^ | 5 | 500 |
| XG5 | t(11;14) | TP53^mut^ | 5 | 360 |
| KMS12PE | t(11;14) | TP53^mut^ | 15 | 200 |
| XG11 | t(11;14) | TP53^mut^ | 2500 | 200 |
| XG1 | t(11;14) | TP53^mut^ | 7000 | 16 |
| U266 | t(11;14) | TP53^mut^ | 8000 | 340 |
| KMM1 | t(6;14) | TP53^mut^ | 5000 | 80 |
| NAN8 | t(4;14) | TP53^trunc^ | 3200 | 350 |
| NCI-H929 | t(4;14) | TP53^WT^ | 5000 | 6 |
| OPM2 | t(4;14) | TP53^mut^ | 5000 | 30 |
| LP1 | t(4;14) | TP53^mut^ | 6000 | 220 |
| JIM3 | t(4;14) | TP53^mut^ | 10 000 | 300 |
| NAN3 | t(4;14) | TP53^mut^ | 5000 | 45 |
| NAN1 | t(14;16) | TP53^trunc^ | 800 | 10 |
| JJN3 | t(14;16) | TP53^neg^ | 1500 | 75 |
| L363 | t(20;22) | TP53^trunc^ | 3000 | 15 |
| XG6 | t(16;22) | TP53^WT^ | 3000 | 25 |
| BCN | t(14;16) | TP53^WT^ | 4000 | 120 |
| MM1S | t(14;16) | TP53^WT^ | 4000 | 200 |
| NAN11 | t(14;16) | TP53^WT^ | 4000 | 10 |
| AMO1 | t(12;14) | TP53^WT^ | 3000 | 3 |
| XG2 | t(12;14) | TP53^mut^ | 3000 | 18 |
| XG10 | t(14;?) | TP53^WT^ | 3400 | 9 |
| XG3 | none | TP53^WT^ | 3500 | 10 |

Supplementary Table 1 : Sensitivity of HMCLs to S63845 and venetoclax. Cell death was assessed by flow cytometry after 24h hours of treatment with increasing concentration of S63845 (0.001-1µM). LD50 values were calculated from 3 independent experiments. LD50 values for venetoclax were previously determined^6,9^
