# Supplementary material for: Dual targeting of BCL2 and MCL1 rescues myeloma cells resistant to BCL2 and MCL1 inhibitors associated with the formation of BAX/BAK hetero-complexes
Source: Cell Death Dis. 2020 May 5;11(5):316. doi: 10.1038/s41419-020-2505-1 (PMC7200824; doi:10.1038/s41419-020-2505-1)
Supplement: Supplementary file 3 — Supplementary Table 2 [file 41419_2020_2505_MOESM3_ESM.docx]

Supplementary Table 2

| # | Log2 *BCL2L1*  mRNA |
| --- | --- |
| 2 | 2.5 |
| 3 | 3.3 |
| 5 | 2.8 |
| 13 | 0.7 |
| 18 | 2.4 |
| 19 | 0.7 |
| 20 | 1.8 |
| 21 | 0.7 |
| 23 | 2.6 |
| 25 | 1.6 |
| 26 | 3.0 |
| 33 | 3.5 |
| 35 | 2.6 |
| 36 | 3.3 |
| 37 | 3.0 |
| 39 | 2.1 |
| 40 | 2.6 |
| 41 | 4.9 |
| 42 | 2.8 |
| 44 | 2.9 |
| 45 | 2.2 |
| 47 | 1.7 |
| 49 | 4.0 |
| 50 | 3.2 |
| 52 | 2.7 |
| 53 | 2.7 |
| 57 | 2.9 |
| 58 | 2.1 |
| 59 | 1.9 |

Table S2 : BCLXL mRNA expression in primary myeloma samples. Among the 60 patients studied for BH3 mimetic sensitivity, CD138+ MM cells from 29 patients have been purified. The levels of BCL2L1 (BCLXL) was generated by DGEseq RNA-sequencing and expressed as log2 normalized mRNA counts.
