# Supplementary figures and images for: Dual targeting of BCL2 and MCL1 rescues myeloma cells resistant to BCL2 and MCL1 inhibitors associated with the formation of BAX/BAK hetero-complexes
Source: Cell Death Dis. 2020 May 5;11(5):316. doi: 10.1038/s41419-020-2505-1 (PMC7200824; doi:10.1038/s41419-020-2505-1)

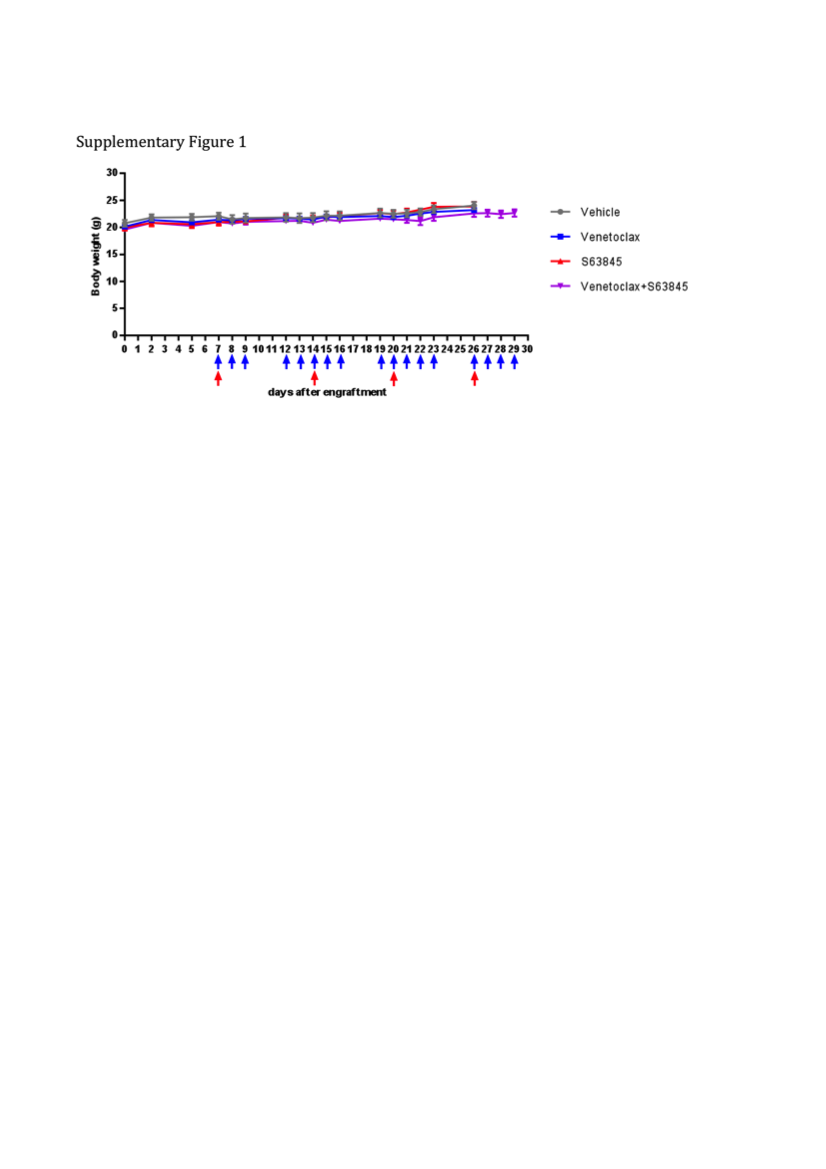

Supplement: Supplementary file 4 — Supplementary Figure 1 [file 41419_2020_2505_MOESM4_ESM.tif]

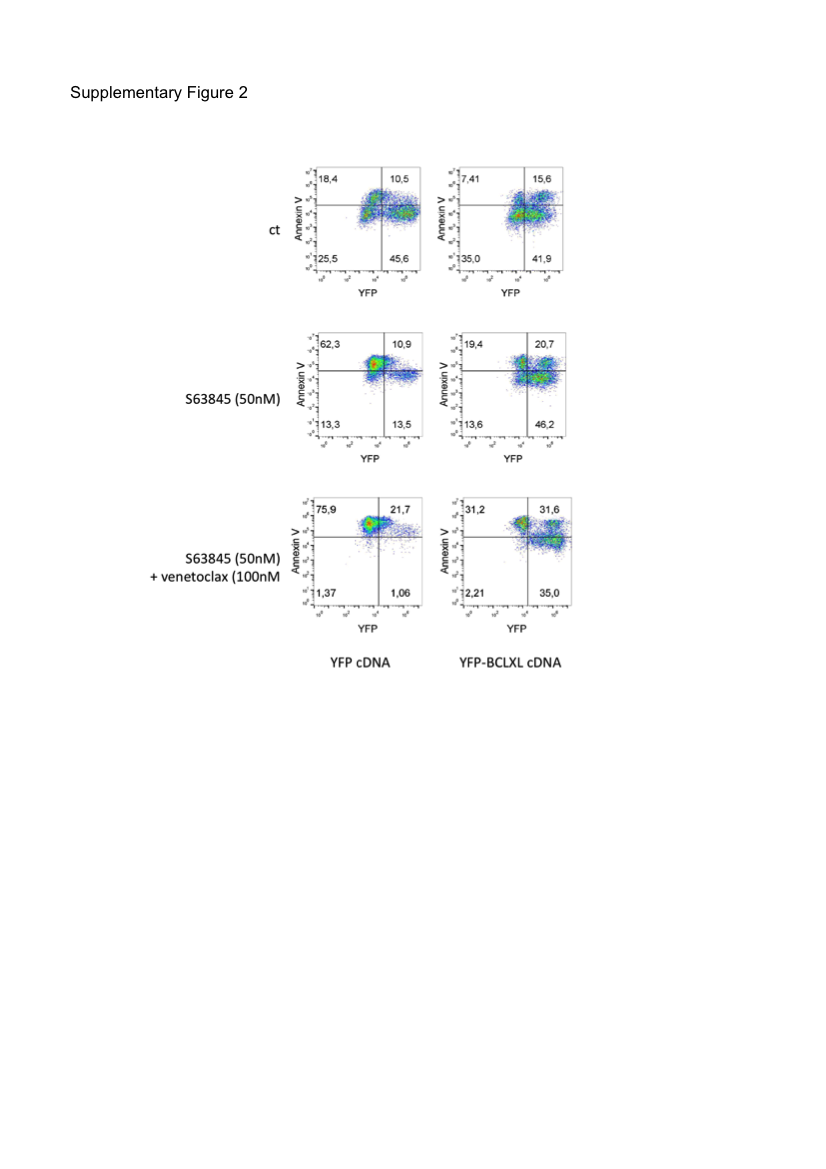

Supplement: Supplementary file 5 — Supplementary Figure 2 [file 41419_2020_2505_MOESM5_ESM.tif]
